# The International Encyclopædia of Surgery

**Published:** 1885-06

**Authors:** 


**The International Encyclopædia of Surgery.** A Systematic Treatise on the Theory and Practice of Surgery by Authors of Various Nations. Edited by JOHN ASHHURST, Jr , M. D., Professor of Clinical Surgery, University of Pennsylvania Illustrated with Chromo-Lithographs and Wood Cuts. In six volumes. Volume v. New York: Wm. Wood & Co. 1884.

This great work has now so nearly reached completion, that the profession can estimate it at its true value. We think that every one who is familiar with it, will say that both editors and publishers have more than fulfilled their promises. For the special purpose for which it is planned, we think there is no work, in any language, which can surpass it. The present volume opens with an article on Injuries of the Head, by Prof. Nancrede; Malformation and Diseases of the Head are well discussed by Dr. Treves, of the London Hospital.

The Injuries and Diseases of the Eyes and their appendages, and of the Ear and Nose and its accessory diseases, are discussed by American experts. Prof. Past's article on the Injuries and Diseases of the Face, Cheek and Lips, supplemented as it is by Christopher Heath, who writes upon the Mouth, Fauces, Tongue, Palate and Jaws, is both comprehensive and able. The Surgery of the Teeth and adjacent parts is followed by articles respectively upon the Injuries and Diseases of the Neck, Air Passages and Abdomen. Prof. Annandale, of University of Edinburg, provides an able article upon Diseases of the Breast. The volume is fully up to the preceding ones, not only in the merit of the articles, but also in the publishers' work, which is, in every way, excellent.

